# The Fanconi anemia pathway controls oncogenic response in hematopoietic stem and progenitor cells by regulating PRMT5-mediated p53 arginine methylation

**DOI:** 10.18632/oncotarget.11088

**Published:** 2016-08-05

**Authors:** Wei Du, Surya Amarachintha, Ozlem Erden, Andrew Wilson, Qishen Pang

**Affiliations:** ^1^ Division of Experimental Hematology and Cancer Biology, Cincinnati, 45229 Ohio, USA; ^2^ Divisions of Radiation Health,College of Pharmacy, UAMS, Little Rock, 72205 Arkansas, USA; ^3^ Department of Pediatrics, University of Cincinnati College of Medicine, Cincinnati, 45229 Ohio, USA

**Keywords:** fanconi anemia, hematopoietic stem and progenitor cells, oncogenic stress, protein arginine methyltransferase 5 (PRMT5)

## Abstract

The Fanconi anemia (FA) pathway is involved in DNA damage and other cellular stress responses. We have investigated the role of the FA pathway in oncogenic stress response by employing an *in vivo* stress-response model expressing the *Gadd45β*-luciferase transgene. Using two inducible models of oncogenic activation (LSL-K-ras^G12D^ and Myc^ER^), we show that hematopoietic stem and progenitor cells (HSPCs) from mice deficient for the FA core complex components Fanca or Fancc exhibit aberrant short-lived response to oncogenic insults. Mechanistic studies reveal that FA deficiency in HSPCs impairs oncogenic stress-induced G_1_ cell-cycle checkpoint, resulting from a compromised K-ras^G12D^-induced arginine methylation of p53 mediated by the protein arginine methyltransferase 5 (PRMT5). Furthermore, forced expression of PRMT5 in HSPCs from LSL-K-ras^G12D^/CreER-*Fanca^−/−^* mice prolongs oncogenic response and delays leukemia development in recipient mice. Our study defines an arginine methylation-dependent FA-p53 interplay that controls oncogenic stress response.

## INTRODUCTION

Fanconi anemia (FA) is a rare inherited disease with at least 19 complementation groups identified thus far [1–7]. Mutations in any of the nineteen FA genes (*FANCA-T*) leads to clinical manifestations characterized by developmental abnormalities, progressive bone marrow failure and a high risk of developing cancer [1–3, 8]. The prominent role of the FA protein family has been implicated in the network of DNA damage response (DDR), which includes DNA repair pathways such as homologous recombination (HR) and non-homologous end-joining (NHEJ) [5, 9–12]. It is established that eight of FA proteins form a FA “core complex” (FANCA-C, E-G, L, and M), which catalyzes the monoubiquitylation of the FANCD2 and FANCI proteins, and activation of downstream DNA repair processes [1–3].

In response to oncogenic activation, normal cells induce genetically encoded programs, mainly growth arrest, apoptosis and senescence, which prevent deregulated proliferation and thus protect multicellular organisms from cancer progression [13–15]. It is known that oncogene-driven proliferation must be associated with inhibition of apoptosis and senescence to allow malignant outgrowth [16–18]. It has been postulated that functional loss of FA proteins renders cells a high predisposition to cancer transformation [1–3, 8, 19, 20]. However, how cells deficient in the FA pathway respond to oncogenic stress remains largely unknown.

The tumor suppressor p53 is a key component of the DNA damage response (DDR) network that activates vital damage containment procedures to restrict aberrant cell growth in response to DNA damage, oncogene activation, and loss of normal cell contacts by maintaining the balance between cell survival and apoptosis [21–24]. It is known that oncogene insults activate p53 [24, 25]. Highly regenerative tissues, such as blood, possess effective DNA damage responses (DDR) that balance long-term regeneration with protection from leukemogenesis [26]. Several recent studies using mouse models suggest a critical role for the p53 pathway in hematopoietic stem cell (HSC) self-renewal and quiescence [27–31] and precise regulation of p53 activity is likely to be important in determining the response of HSCs to DNA damage and oncogenic activation. Insufficient p53 activation would favor cell survival, but put cells at risk for loss of genomic integrity. In contrast, excessive p53 activation could compromise steady-state hematopoiesis and its recovery following exogenous marrow insult by causing too many cells to be eliminated [32]. In the context of FA, emerging evidence suggests that p53 deficiency may increase cancer development in patients with FA and FA mice [33–36]. Conversely, recent studies show that overactive p53 could cause hematopoietic stem and progenitor cell (HSPC) depletion in the BM of FA patients [37]. These studies corroborate a critical role of the FA proteins in cooperating with p53 in apoptosis and cell cycle checkpoint control after DNA damage induced by an exaggerated physiologic stress response.

In the present study, we employed an *in vivo* stress-response model expressing the luciferase transgene under the control of the promoter of the stress-responsive gene *Gadd45β* and showed that HSPCs from mice deficient for the core complex components of the FA pathway, Fanca or Fancc, exhibited aberrant response to oncogenic stress. In a sharp contrast to wild-type controls, *Fanca^−/−^* or *Fancc^−/−^* HSPCs showed a short-lived response to oncogenic activation. Significantly, we demonstrated that disruption of the FA pathway compromised the oncogene K-ras^G12D^-induced arginine methylation of p53 mediated by the protein arginine methyltransferase 5 (PRMT5). Therefore, our study demonstrates for the first time that oncogenic stress orchestrates a p53-dependent response that is controlled by PRMT5-mediated arginine methylation and identifies the FA pathway as an integral part of this versatile cellular mechanism.

## RESULTS

### Disruption of the FA pathway induces a short-lived response to oncogenic stress *in vitro*

**Figure 1:**
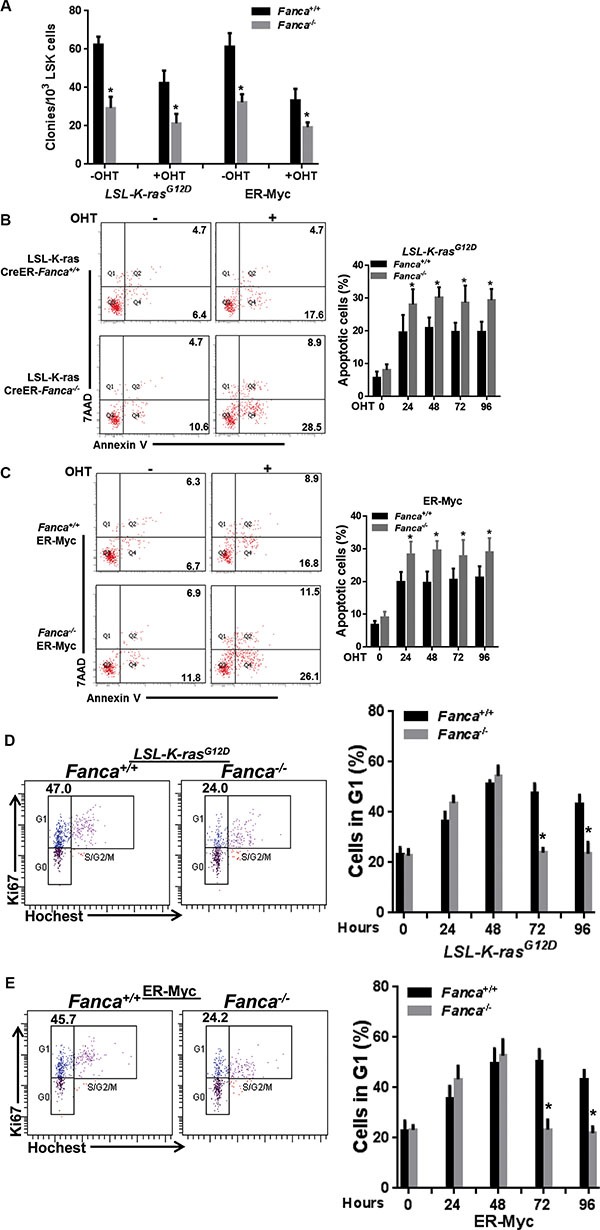
Disruption of the FA pathway induces a short-lived response to oncogenic stress *in vitro* (**A**) Oncogenic stress compromises colony formation capacity of FA HSPCs. LSK cells (Lin^−^Sca1^+^c-kit^+^ cells) isolated from LSL-*Fanca^+/+^/*K-ras/CreER and LSL-*Fanca^−/−^/*K-ras/CreER mice, or retroviral vector MSCV-IRES-Myc^ER^ transduced LSK cells from *Fanca^+/+^* or *Fanca^−/−^* mice were *in vitro* culture in the presence of 4-OHT for 48 hours followed by plating in cytokine-supplemented methycellulose medium. Colonies were enumerated on day 7 after plating. Results are means ± standard deviation (SD) of 3 independent experiments (*n* = 9 per group). (**B**) K-ras activation induces apoptosis in FA HSCs. LSK cells (Lin^−^Sca1^+^c-kit^+^ cells) isolated from LSL-*Fanca^+/+^/*K-ras/CreER and LSL-*Fanca^−/−^/*K-ras/CreER mice were subjected to Flow cytometric analysis for apoptosis by Annexin V/7AAD staining at different time points. Representative images at time 0 and 24 h after *4*-*OHT* induction (left) and quantification (right) were shown. Results are means ± standard deviation (SD) of 3 independent experiments (*n* = 6 per group). (**C**) Myc activation induces apoptosis in FA HSCs. Retroviral vector MSCV-IRES-Myc^ER^ transduced LSK cells from *Fanca^+/+^* or *Fanca^−/−^* mice were subjected to Flow cytometric analysis for apoptosis by Annexin V/7AAD staining at different time points. Representative images at time 0 and 24 h after *4*-*OHT* induction (left) and quantification (right) were shown. Results are means ± standard deviation (SD) of 3 independent experiments (*n* = 9 per group). (**D**) Activation of K-ras leads to short-lived G_1_ arrest in FA cells. Cells described in (B) were cultured in the presence of 4-OHT for 2 hours then released in fresh medium for the indicated time intervals, followed by cell cycle profiling by Hochest33324/Ki67 staining. Representative images (left) and quantification (right) were shown. Results are means ± standard deviation (SD) of 3 independent experiments (*n* = 6 per group). (**E**) Activation of Myc leads to short-lived G_1_ arrest in FA cells. Cells described in (C) were cultured in the presence of 4-OHT for 2 hours then released in fresh medium for the indicated time intervals, followed by cell cycle profiling by Hochest33324/Ki67 staining. Representative images (left) and quantification (right) were shown. Results are means ± standard deviation (SD) of 3 independent experiments (*n* = 9 per group).

To investigate how cells with a defective FA pathway respond to oncogenic stress, we employed two inducible models of oncogenic activation: 1) the LSL-K-ras^G12D^/CreER mouse model [38], in which Cre-mediated recombination leads to deletion of a translational termination sequence (Lox-Stop-Lox) and expression of the oncogenic Kras^G12D^ protein; 2) A retroviral vector delivered proto-oncogene Myc^ER^ system [39], in which oncogene activation is induced and controlled by 4-OHT or tamoxifen exposure. The reason that we chose the K-ras^G12D^ model for these detailed studies was two-fold: 1) it is an authentic *in-vivo* knock-in model, which enabled us to analyze oncogenic response under near physiological conditions; and 2) it is an established myeloid leukemia model, which has relevance to FA disease progression. We first examined the sensitivity of hematopoietic stem and progenitor (HSPC; LSK) cells (Figure S1A), isolated from LSL-K-ras^G12D^/CreER mice or infected with the Myc^ER^ retrovirus, to oncogene activation by *in vitro* culturing the cells in the presence of 4-Hydroxytamoxifen (*4*-*OHT*) for 48 hours [40, 41] followed by plating in cytokine-supplemented methycellulose medium. We found that K-ras or Myc activation reduced colony formation in both WT and *Fanca^−/−^* or *Fancc^−/−^* progenitors (Figures 1A, S1B), which accompanied by increased apoptosis 24–96 h after *4*-*OHT* induction (Figures 1B, 1C, S1C, S1D).

To determine the kinetics of oncogenic response, we assessed G_1_ cell cycle arrest induced by K-ras or Myc activation [42, 43]. Hochest 33342/Ki67 staining showed significantly increased percentage of LSK cells arrested in G_1_ phase in both WT and *Fanca^−/−^* or *Fancc^−/−^* after 4-OHT treatment (Figures 1D, 1E, S1E, S1F). However, oncogenic activation of K-ras or Myc induced prolonged G_1_ arrest in WT LSK cells (Figures 1D, 1E, S1E, S1F). In contrast, *Fanca^−/−^* or *Fancc^−/−^* LSK cells showed a short-lived G_1_ arrest with a peak at 48 hours and returned to cycle at 72 hours after 4-OHT induction (Figures 1D, 1E, S1E, S1F). These *in vitro* results demonstrate an aberrant short-lived oncogenic stress response in FA HSPCs.

### Disruption of the FA pathway induces a short-lived response to oncogenic stress *in vivo*

**Figure 2:**
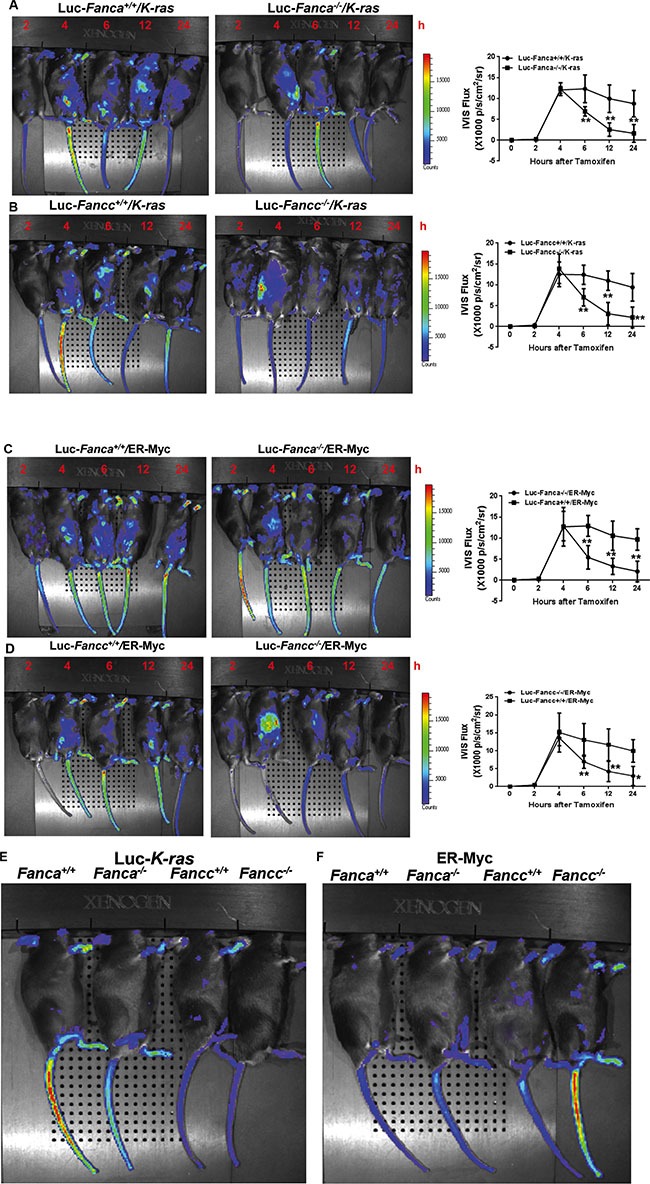
Disruption of the FA pathway induces a short-lived response to oncogenic stress *in vivo* (**A**, **B**) FA mice exhibit short-lived response to K-ras activation. 1,000 LSK cells from Luc-LSL-K-ras/CreER-*Fanca^+/+^* or 2,000 LSK cell from Luc-LSL-K-ras/CreER-*Fanca^−/−^* mice (A) along with 3 × 10^5^ BM cells from congenic BoyJ mice were transplanted into lethally irradiated BoyJ recipients. 4-month post BMT, the recipients were i.p injected with single dose of Tamoxifen followed by IVIS imaging at the indicated time points. Luminescence scale is in p/s/cm2/sr. Similar experiments were conducted on *Fancc^−/−^* mice (B). (**C**, **D**) FA mice exhibit short-lived response to Myc^ER^ activation. 1,500 retroviral vector MSCV-IRES-Myc^ER^ transduced Luc-*Fanca^+/+^* cells or 3,000 transduced Luc-*Fanca^−/−^* cells (C) along with 3 × 10^5^ BM cells from congenic BoyJ mice from recipient mice were transplanted into lethally irradiated BoyJ recipients. 4-month post BMT, the recipients were i.p injected with single dose of Tamoxifen followed by IVIS imaging at the indicated time points. Luminescence scale is in p/s/cm2/sr. Similar experiments were conducted on *Fancc^−/−^* mice (D). The bioluminescent image signals were quantified using LiveImage Pro. 2.0 software. Results are means ± standard deviation (SD) of 3 independent experiments (*n* = 6 per group). (**E**, **F**) Minimum bioluminescence of Luc mice without tamoxifen injection (the “0” time-point controls). Luminescence scale is in p/s/cm2/sr.

We next examined oncogenic response *in vivo* by crossing the FA mice to the Luc-*Gadd45β* mice, which express the luciferase transgene under the control of the promoter of the stress-responsive gene *Gadd45β* [44] and allow for non-invasive *in vivo* imaging stress-induced expression of the luciferase marker. Gadd45*β* is well established for its diverse roles in cell cycle control, cell survival, apoptosis, DNA damage repair and the maintenance of genomic stability [45]. Gadd45*β* can also act as a stress sensor in the development of hematopoietic malignancies like leukemia [46]. We assessed oncogenic response in the recipients with similar donor chimerism (Figure S2), transplanted with BM LSK cells expressing Luc-*Gadd45β* and LSL-K-ras^G12D^/CreER or Myc^ER^, by monitoring the kinetics of luciferase expression at different time points after tamoxifen injection. Consistent with the *in vitro* observations described above, the recipients transplanted with WT (*Fanca^+/+^* or *Fancc^+/+^*) LSK cells expressing LSL-K-ras^G12D^/CreER (Figure 2A, 2B, 2E) or Myc^ER^ (Figure 2C, 2D, 2F) exhibited a persistent induction of luciferase expression even 48 hours after tamoxifen injection. In contrast, the recipients transplanted with FA-deficient (*Fanca^−/−^* or *Fancc^−/−^*) LSK cells expressing LSL-K-ras^G12D^/CreER (Figure 2A, 2B, 2E) or Myc^ER^ (Figure 2C, 2D, 2F) showed a typical short-lived response with a peak at 4 hours and declined from 6–24 hours after induction. Taken together, HSPCs deficient for the *Fanca* or *Fancc* gene displayed abnormal short-lived response to oncogenic stress. In addition, we observed a similar pattern of less long-lived oncogenic response in primary mouse embryonic fibroblasts (MEFs) isolated from Luc-LSL-K-ras/CreER-*Fanca^−/−^* mice (Figure S3), suggesting that this abnormal oncogenic response is not restricted to HSPCs.

### Deregulated G_1_ checkpoint regulators correlated with short-lived oncogenic response

**Figure 3:**
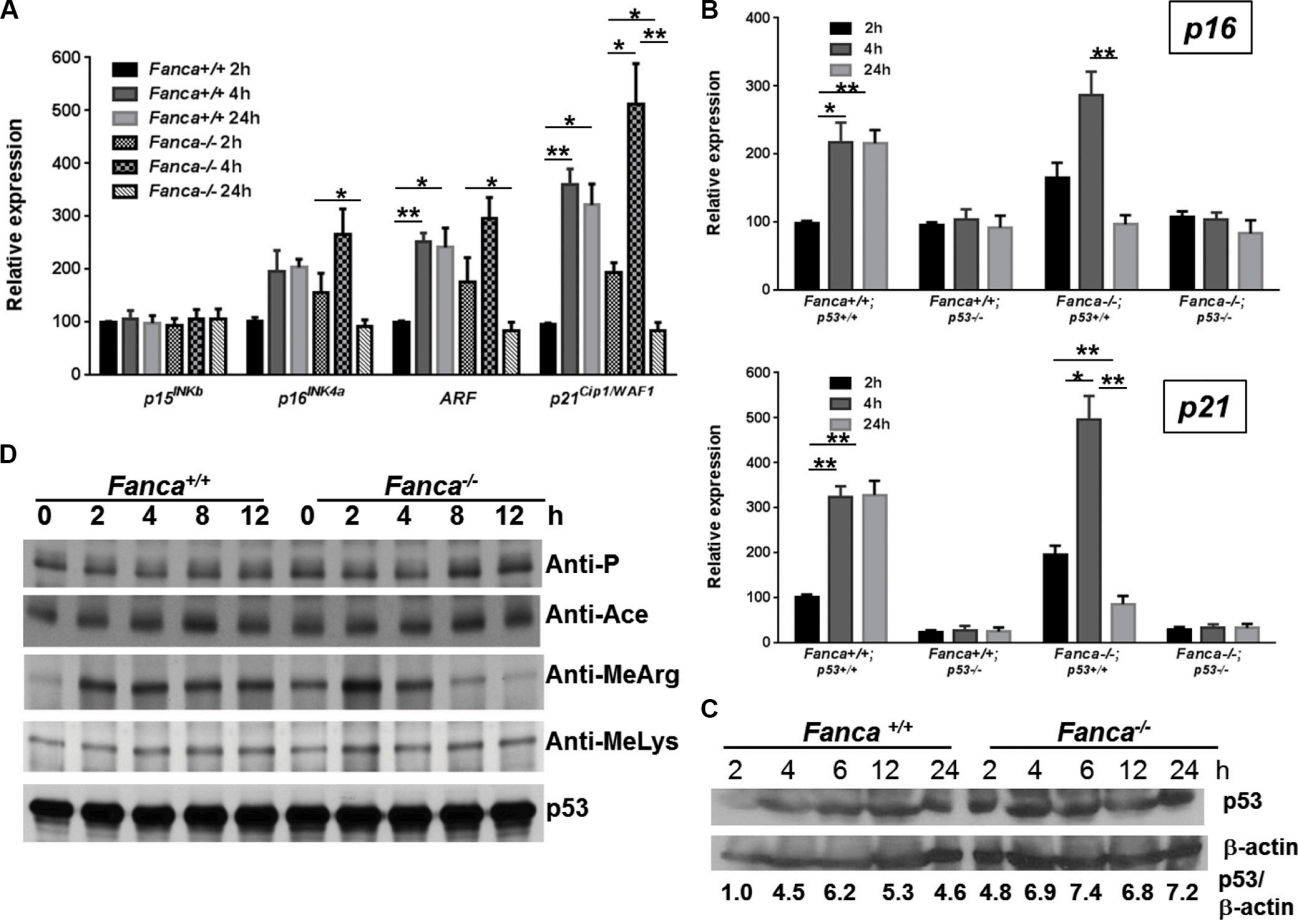
Aberrant oncogene-induced arginine methylation of p53 contributes to short-lived oncogenic response (**A**) Short-lived response of G_1_-arrest-associated cell-cycle regulators in response to oncogenic stress in FA HSPCs. LSK cells isolated from Luc-LSL-*Fanca^+/+^/*K-ras/CreER or Luc-LSL-*Fanca^−/−^/*K-ras/CreER mice were cultured in the presence of 4-OHT for 2 hours then released in fresh medium for the indicated time intervals. RNA was extracted at different time points for qPCR analysis using primers listed in Table S1. Levels of the expression in each sample were normalized to the level of *GAPDH* mRNA, and the expression levels of the *Fanca^+/+^* samples at 2 h were normalized as 100. (**B**) p53 is essential for the prolonged oncogenic response in HSPCs. LSK cells isolated from LSL-*Fanca^+/+^*/K-ras/CreER-*p53^+/+^*, LSL-*Fanca^−/−^*/K-ras/CreER-*p53^+/+^,* LSL-*Fanca^+/+^*/K-ras/CreER-*p53^−/−^*, LSL-*Fanca^−/−^*/K-ras/CreER-*p53^−/−^* mice were cultured in the presence of 4-OHT for 2 hours then released in fresh medium for the indicated time intervals. RNA was extracted at different time points for qPCR analysis using primers for *p16* (Upper) and *p21* (Lower) Samples were normalized to the level of *GAPDH* mRNA. (**C**) K-ras activation does not alter p53 level. BM Lin^−^ cells from Luc-LSL-*Fanca^+/+^/*K-ras/CreER or Luc-LSL-*Fanca^−/−^/*K-ras/CreER mice were cultured in the presence of 4-OHT for 2 hours then released in fresh medium for the indicated time intervals. Whole cell lysates were prepared then subjected to immunoblot using antibodies against p53 or β-actin. p53:β-actin ratio in each sample was calculated. The WT control sample at 0 h time point has been normalized as 1. (**D**) FA deficiency leads to short-lived arginine methylation of p53 in response to oncogene activation. BM Lin^−^ cells from Luc-LSL-*Fanca^+/+^/*K-ras/CreER or Luc-LSL-*Fanca^−/−^/*K-ras/CreER mice were cultured in the presence of 4-OHT for 2 hours then released in fresh medium for the indicated time intervals. Whole cell lysates were prepared, and subjected to immunoprecipitation using anti-p53 antibody followed by immunoblotting with antibodies against phosphor-serine, acetylated lysine, methylated lysine, mono-methyl arginine or p53, respectively.

The observation that *Fanca^−/−^* and *Fancc^−/−^* HSPCs exhibited an abnormal short-lived response to K-ras and c-Myc activation prompted us to investigate the underlying molecular mechanism. Since activation of oncogenes including K-ras and c-Myc induces senescence-like cell growth arrest in a variety of cell types [47, 48] and since we observed a short-lived G_1_ arrest induced by K-ras or c-Myc activation in *Fanca^−/−^* and *Fancc^−/−^* LSK cells (Figures 1D, 1E, S1E, S1F), we analyzed oncogene-induced expression of G_1_ arrest-associated cell-cycle regulators in K-Ras^G12D^-expressing HSPCs with a focus on *p15^INKb^*, *p16^INK4a^*, *ARF and p21^Cip1/WAF1^* [49, 50]. Indeed, it is known that the K-ras^G12D^ mutation is frequently associated with juvenile myelomonocytic leukemia (JMML) and acute myeloid leukemia (AML) [51]. LSK cells freshly isolated from WT and *Fanca^−/−^* littermates expressing the LSL-K-ras^G12D^/CreER transgenes were treated with 4-OHT at different time points to determine the kinetics of oncogenic induction of the G_1_ arrest-associated cell-cycle regulators. Interestingly, three of the four G_1_ arrest-associated cell-cycle regulators, namely *p16^INK4a^*, *p19^Arf^* and *p21^Cip1/WAF1^*, exhibited the pattern of short-lived response to K-ras activation in *Fanca^−/−^* HSPCs. That is, K-ras activation orchestrated the expression of these cell-cycle regulators that burst at 4 hours then receded to the basal level at 24 hours (Figure 3A). In contrast, the WT LSK cells displayed a persistent response during the 4–24 hours period (Figure 3A). These results suggest that a pathway regulating the expression of *p16^INK4a^*, *p19^Arf^* and *p21^Cip1/WAF1^* may be responsible for the short-lived oncogenic stress response in *Fanca^−/−^* HSPCs.

### Short-lived oncogenic response involves p53 signaling

Since *p21^Cip1/WAF1^* is a p53 target and *p19^Arf^* induction by K-ras requires p53 [49, 50, 52], we determined if p53 was essential for the prolonged oncogenic response in HSPCs. We deleted the *Trp53* gene in our *Fanca^−/−^* LSL-K-ras^G12D^/CreER mice, and evaluated the requirement of p53 for K-ras-induced OIS (oncogene-induced senescence) [53] using *p16^INK4a^* expression as a surrogate. As illustrated in Figure 3B, loss of p53 not only abrogated the long-lasting (4–24 hours) K-ras-induced *p16^INK4a^* expression in WT LSL-K-ras^G12D^/CreER LSK cells but also abolished the short-lived (4 hours) response to K-ras activation in *Fanca^−/−^* LSL-K-ras^G12D^/CreER LSK cells (Figure 3B, Upper). Consistently, p53 inactivation completely abated *p21^Cip1/WAF1^* expression in response to K-ras activation in both WT and *Fanca^−/−^* LSL-K-ras^G12D^/CreER LSK cells (Figure 3B, Lower). Thus, an alteration in p53 protein level or activity during the 4–24 hours period may account for the short-lived oncogenic response in *Fanca^−/−^* LSL-K-ras^G12D^/CreER LSK cells.

### Aberrant oncogene-induced arginine methylation of p53 contributes to short-lived oncogenic response

We next examined the status of p53 in WT and *Fanca^−/−^* LSL-K-ras^G12D^/CreER HSPCs. While *Fanca^−/−^* BM lineage-negative (Lin^−^) cells (enriched for HSPCs) showed higher levels of p53 protein than WT cells, we did not observe significant difference in the kinetics of p53 induction between WT and *Fanca^−/−^* cells during the 2–24 hours period of K-ras activation (Figure 3C). This suggests the aberrant oncogenic response observed in *Fanca^−/−^* HSPCs was most likely resulted from an altered p53 activity. Since most if not all of the p53 activities are controlled by post-translational modifications [54], we determined phosphorylation, acetylation and methylation of p53 in WT and *Fanca^−/−^* LSL-K-ras^G12D^/CreER HSPCs. The p53 protein was enriched by immunoprecipitation using lysates of BM Lin^−^ cells treated with 4-OHT for different time intervals, and probed for forms of p53 modified by phosphorylation, acetylation or methylation under oncogenic stress. We found similar levels of phosphorylation, acetylation, or lysine methylation of p53 between WT and *Fanca^−/−^* cells at different time points after oncogene induction (Figure 3D). However, K-ras-induced arginine methylation of p53 was peaked at 2 hours and almost completely abated after 8-hour induction with 4-OHT in *Fanca^−/−^* Lin^−^ cells (Figure 3D), reminiscent of the short-lived oncogenic response described above. Thus, these results identified an aberrant oncogene-induced arginine methylation of p53 as a potential mechanism for the short-lived oncogenic stress response in FA HSPCs.

### Ectopic expression of PRMT5 prevents short-lived oncogenic response

**Figure 4:**
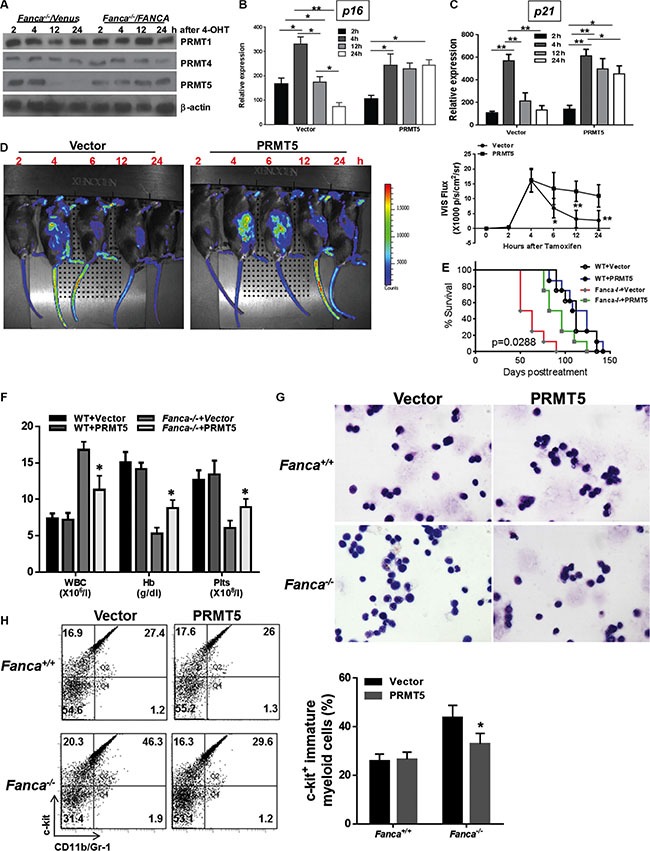
PRMT5-mediated arginine methylation of p53 prevents short-lived oncogenic response (**A**) Progressive decline of PRMT5 in FA HSPCs in response to oncogenic stress. Lin^−^ cells isolated from Luc-LSL-*Fanca^+/+^/*K-ras/CreER or Luc-LSL-*Fanca^−/−^/*K-ras/CreER mice were transduced with retroviral vector expressing Venus or Venus/*FANCA*. Sorted Venus^+^ cells were then cultured in the presence of 4-OHT for 2 hours then released in fresh medium for the indicated time intervals. Whole cell lysates (WCL) was then extracted from the transduced cells for immunoblotting using antibodies against PRMT1, PRMT4, PRMT5 and β-actin. (**B**, **C**) Overexpression of PRMT5 abrogates the short-lived K-ras-induced *p16^INK4a^* and *p21^Cip1/WAF1^* expression in FA HSPCs. Lin^−^ cells isolated from Luc-LSL-*Fanca^−/−^/*K-ras/CreER mice were transduced with lentiviral vector expressing eGFP or eGFP/PRMT5. Sorted eGFP^+^ LSK cells were cultured in the presence of 4-OHT for 2 hours then released in fresh medium for the indicated time intervals, followed by RNA extraction and qPCR analysis using primers for *p16* (B) and *p21^Cip1/WAF1^*(C). Samples were normalized to the level of *GAPDH* mRNA. (**D**) Overexpression of PRMT5 lengthens the oncogenic response. Cells described in (B) were transplanted into lethally irradiated BoyJ recipients. 4 months post BMT, single dose of tamoxifen was injected to the recipient animals followed by IVIS imaging at different time points. Luminescence scale is in p/s/cm2/sr. The bioluminescent image signals were quantified using LiveImage Pro. 2.0 software. Results are means ± standard deviation (SD) of 3 independent experiments (*n* = 6 per group). (**E**) Ectopic PRMT5 expression delays leukemia development. Survival of recipients described in (D) was monitored and plotted by the Kaplan-Meier curve method (*n* = 19 per group). The difference in the recipients transplanted with *Fanca^−/−^* plus vector versus *Fanca^−/−^* plus PRMT5 reaches a *p value* of 0.0288 (Log-rank test). Controls without tamoxifen (the “0” time-point controls) are depicted in Supplementary Figures. (**F**) Blood count of the leukemic mice. Peripheral blood from the mice described in (D) was subjected to blood count using HemaVet 950. Quantifications are shown (*n* = 6 per group). (**G**) Wright-Gimesa staining of bone marrow. Bone marrow samples from the recipients described in (D) were subjected to Wright-Gimesa staining. Representative images are shown. (**H**) Increased infiltration of myeloid blasts in spleen. Splenocytes from the recipients described in (D) were subjected to Flow cytometry analysis for myeloid blast. Representative flow plots (Left) and quantifications (Right) are shown (*n* = 6 per group).

Protein arginine methylation is mediated by a group of protein arginine methyltransferases (PRMTs) [55]. Three of the PRMTs (PRMT1, 4 and 5) have been implicated in regulating p53-mediated transcription [50–52]. We thus examined the levels of these PRMTs in *Fanca^−/−^* LSL-K-ras^G12D^/CreER Lin^−^ cells transduced with Venus or Venus-*FANCA* lentivirus and then subjected to oncogenic stress. We found that the level of PRMT5 underwent progressive decline after 4 hours of 4-OHT induction in *Fanca^−/−^* LSL-K-ras^G12D^/CreER Lin^−^ cells, and re-expression of the *FANCA* gene in these cells stabilized the PRMT5 protein during the 24-hour period of 4-OHT induction (Figure 4A).

The correlation between aberrant oncogene-induced arginine methylation of p53 accompanied by a progressive decline in PRMT5 and the short-lived oncogenic stress response in FA HSPCs prompted us to determine whether restoring the level of PRMT5 could lengthen the oncogenic response in FA HSPCs. To this end, we ectopically expressed GFP or GFP-PRMT5 in WT and *Fanca^−/−^* Lin^−^ cells expressing the Luc-LSL-K-ras^G12D^/CreER cassette by lentiviral transduction (Figure S4A). The transduced (GFP^+^) cells were then sorted for LSK cells and used for analyzing oncogenic response *in vitro* and *in vivo*. First, we analyzed the sorted GFP^+^ LSK cells for the kinetics of *p16^INK4a^* expression at different time-points of K-ras^G12D^ induction following 4-OHT treatment. We found that ectopic expression of PRMT5 completely abrogated the short-lived K-ras-induced *p16^INK4a^* expression in *Fanca^−/−^* LSK cells (Figure 4B). Lentiviral expression of PRMT5 did not have effect on K-ras-induced *p16^INK4a^* expression in WT LSK cells (Figure S4B).

To specifically demonstrate the effect of ectopic expression of PRMT5 on p53 response, we determined the kinetics of oncogene-induced expression of the p53 target *p21^Cip1/WAF1^* using vector- or PRMT5-transduced LSK cells from *Fanca^−/−^* LSL-K-ras^G12D^/CreER mice with WT or p53-null background. As illustrated in Figure 4C, forced expression of PRMT5 showed a prolonged pattern, similar to that seen in WT LSK cells (Figure 3B), of *p21^Cip1/WAF1^* expression in response to K-ras activation in p53-sufficient *Fanca^−/−^* LSL-K-ras^G12D^/CreER LSK cells but not in p53-defficient *Fanca^−/−^* LSL-K-ras^G12D^/CreER LSK cells (Figure 4C). This result suggests that p53 is at least one of the targets of ectopically expressed PRMT5 in response to K-ras^G12D^ in thes*e* LSK cells.

### Forced expression of PRMT5 delays leukemia

To determine the effect of ectopic expression of PRMT5 on oncogenic response *in vivo*, we transplanted the transduced (GFP^+^) WT and *Fanca^−/−^* Luc-LSL-K-ras^G12D^/CreER LSK cells into lethally irradiated BoyJ recipients. Four months later, the recipients were treated with tamoxifen and assessed oncogenic response by monitoring the kinetics of luciferase expression at different time points. It appeared that exogenous expression of PRMT5 not only lengthened but also enhanced the activated K-ras response in the progeny of donor *Fanca^−/−^* Luc-LSL-K-ras^G12D^/CreER HSPCs (Figures 4D, S4C). Lentiviral expression of PRMT5 also augmented K-ras-induced luciferase expression in WT donor-derived cells (Figure S4D). Furthermore, K-ras^G12D^-induced leukemia was significantly delayed in recipient mice transplanted with *Fanca^−/−^* Luc-LSL-K-ras^G12D^/CreER LSK cells expressing PRMT5 compared with the recipient mice of donor *Fanca^−/−^* Luc-LSL-K-ras^G12D^/CreER LSK cells carrying the vector control virus (Figure 4E). Further characterization of the leukemic mice showed that the recipient mice transplanted with the *Fanca^−/−^* Luc-LSL-K-ras^G12D^/CreER LSK cells developed myeloid leukemia, as characterized by increased white blood cells (WBCs) in the peripheral blood, anemia, and infiltration of myeloid blasts in the spleen and bone marrow (Figure 4F–4H). Collectively, these results link attenuated PRMT5 activity to the short-lived oncogenic stress response and leukemia development in FA HSPCs.

**Figure 5:**
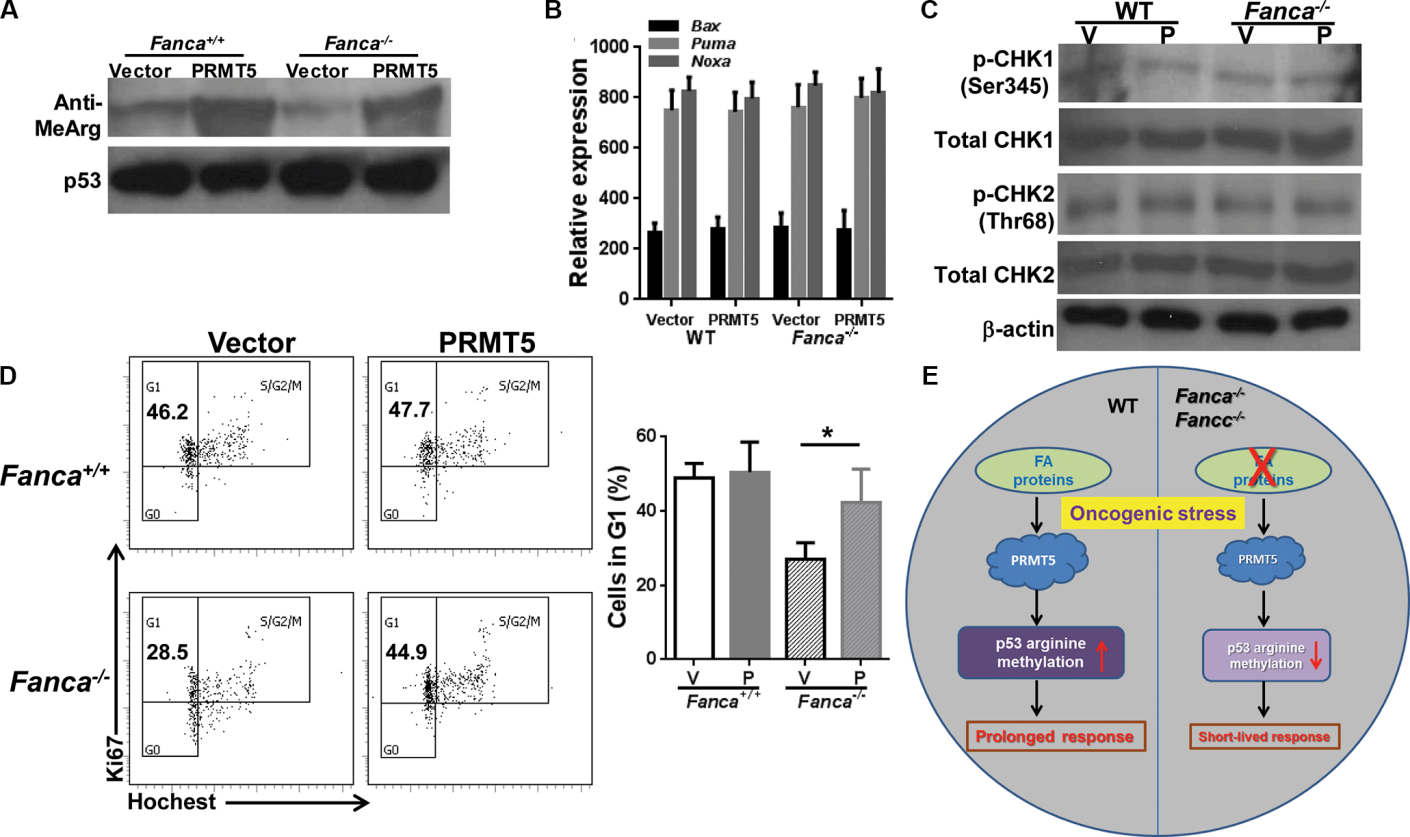
Forced expression of PRMT5 does not affect ATM/ATR pathway (**A**) Ectopic PRMT5 expression increased arginine methylation of p53 in Kras^G12D^-expressing WT and *Fanca^−/−^* leukemic cells. Lin^−^ cells isolated from Luc-LSL-*Fanca^−/−^/*K-ras/CreER mice were transduced with lentiviral vector expressing eGFP or eGFP/PRMT5. Sorted eGFP^+^ LSK cells were transplanted into lethally irradiated BoyJ recipients. 4 months post BMT, single dose of tamoxifen was injected to the recipient animals. Whole cell lysates were extracted from Lin^−^ cells isolated from leukemic mice followed by immunoprecipitation using anti-p53 antibody and probed with antibodies against mono-methyl arginine or p53, respectively. (**B**) Ectopic expression of PRMT5 does not affect apoptotic gene expression. RNA was extracted from cells described in (A) followed by qPCR analysis using primers for *Bax*, *Puma* or *Noxa*. (**C**) Forced expression of PRMT5 does not alter CHK1 or CHK2 phosphorylation. Whole cell lystates extracted from cells described in (B) were subjected to immunoblotting using antibodies against phosphor-CHK1 (Ser245), total CHK1, phosphor-CHK2 (Thr68), total CHK2 or β-actin. (**D**) Forced expression of PRMT5 prolonged the short-lived G_1_ arrest induced by K-ras activation in *Fanca^−/−^* leukemic cells. Cells described in (B) were cultured in the presence of 4-OHT for 2 hours then released in fresh medium for 72 hours followed by cell cycle analysis. Representative images and quantification were shown. (**E**) Model of PRMT5-p53 Arg-methylation in oncogenic response.

We further examined the molecular and cellular effects of forced expression of PRMT5 by analyzing the levels of p53 arginine methylation, the transcriptional activation of p53, the activity of DNA damage response kinases ATM and ATR, and cell cycle status in the leukemic cells. As expected, ectopic PRMT5 expression increased arginine methylation of p53 in K-ras^G12D^-expressing WT and *Fanca^−/−^* leukemic cells (Figure 5A). In contrast to an increased expression of the p53 target gene in cell-cycle control, *p21^Cip1/WAF1^* (Figure 4C), forced expression of PRMT5 did not enhance expression of several major p53 apoptotic target genes like *Bax*, *Puma*, or *Noxa* in these leukemic cells (Figure 5B). PRMT5 expression did not increase the activity of the key DNA damage response kinases ATM and ATR, using phosphorylation of CHK1 and CHK2 as the respective surrogate (Figure 5C). However, forced expression of PRMT5 prolonged the short-lived G_1_ arrest induced by K-ras activation in *Fanca^−/−^* leukemic cells (Figure 5D). These results suggest that PRMT5 may exert its function via the p53/G_1_ cell-cycle checkpoint rather than the ATM/ATR/G_2_ DNA damage checkpoint in our oncogenic response model.

## DISCUSSION

The fact that patients with Fanconi anemia (FA) gene mutations have high predisposition to leukemia and other cancers indicates that FA disease progression involves oncogenic transformation. Little is known about the mechanism by which these mutant cells respond to oncogenic activation. In the present study, we investigated the response of FA HSPCs to oncogene insults and found that the FA pathway plays an important role in response to oncogenic stress. There are several findings that highlight the significance of our study: 1) FA HSPCs displayed an aberrant short-lived response to oncogenic stress induced by activated K-ras or c-Myc; 2) *Fanca* deficiency compromises K-ras^G12D^-induced arginine methylation of p53 accompanied by downregulated PRMT5; 3) forced expression of PRMT5 in *Fanca^−/−^* HSPCs prolonged oncogenic response and delayed leukemia development in irradiated recipient mice. Based on these observations, we propose a model in which the FA proteins in WT cells may play a role in regulating PRMT5-mediated p53 arginine methylation in response to oncogenic stress leading to prolonged oncogenic response. On the other hand, the regulatory function of PRMT5 on p53 is diminished in *Fanca-* or *Fancc-*deficient cells, which leads to decreased p53 arginine methylation and short-live oncogenic response (Figure 5E).

An important and novel finding of the present study is the short-lived response of FA HSPCs to oncogenic insults. It is known that FA patients have an increased susceptibility to cancer including leukemia [1–3, 8, 19, 20]. This may be resulted from increased genomic instability, as cells from FA patients are defective in repair of DNA damage induced by certain genotoxic agents [4, 5, 8, 20]. Genes encoding proteins with cancer-promoting (oncogenes like K-ras and Myc) or suppressive (tumor suppressors like p53 and ATM) activities in FA cells may be vulnerable to alterations due to a deficit in DNA repair. These alterations may create mutations that activate oncogenes or inactivate tumor suppressors leading to cancer transformation. How FA HSPCs respond to oncogene activation has not been studied. Here we employed two oncogenic systems, the LSL-K-ras^G12D^/CreER transgenic mouse model and the inducible Myc^ER^ model, and observed a unique phenomenon featuring a short-lived response of FA HSPCs to oncogene activation both *in vitro* and *in vivo*. Whether this aberrant short-lived response is the consequence of mutations that disrupt oncogenic signaling pathways remains to be investigated.

We have made effort to identify the signaling pathway that is responsible for the observed short-lived oncogenic response in FA HSPCs. Our results implicate a role of the tumor suppressor p53. Several lines of evidence support this notion: first, the kinetics of oncogene-induced expression of p53 downstream signaling targets *p19^Arf^* and *p21^Cip1/WAF1^* displayed the exact pattern of the expression of the *Gadd45β*-luciferase transgene and *p16^INK4a^* in *Fanca^−/−^* HSPCs (Figures 2A, 2B, 2C, 2D and Figure 3A). Second, deletion of the *Trp53* gene abrogated both the long-lasting and the short-lived oncogenic responses in WT and *Fanca^−/−^* LSL-K-ras^G12D^/CreER LSK cells, respectively (Figure 3B, Upper). Third, p53 loss completely abolished *p21^Cip1/WAF1^* expression, which is absolutely required for the survival of HSPCs, in response to K-ras activation in both WT and *Fanca^−/−^* LSL-K-ras^G12D^/CreER LSK cells (Figure 3B, Lower). Reduced level of p21 may lead to increased apoptosis upon oncogene induction and p53/p21 deficiency could simultaneously cause DNA damage and de-regulate cell cycle leading to mitotic catastrophe. These results thus identify the p53 tumor suppressor pathway that interplays with the FA pathway in oncogenic response. In addition, our finding further corroborates the previous reports that loss of p53 increases cancer development in patients with FA and FA knockout mice [34–36].

Our results also suggest that an alteration of p53 protein level or activity during time window of 4–24 hours following K-ras activation might account for the short-lived oncogenic response in *Fanca^−/−^* LSL-K-ras^G12D^/CreER LSK cells. The observation that there was no difference in p53 protein level between WT and *Fanca^−/−^* LSK cells during the 2–24 hours period of K-ras activation (Figure 3C), shifted our attention to an altered p53 activity. In normal cells, cellular stresses including oncogenic activation initiates a signaling network involving enzymes that target p53 in the form of post-translational modifications, such as phosphorylation, acetylation and methylation [54]. Protein arginine methylation is mediated by a group of protein arginine methyltransferases (PRMTs) an important process and involved in the regulation of gene expression, RNA metabolism and protein function [55]. Remarkably, we showed that *Fanca* deficiency compromised K-ras^G12D^-induced arginine methylation of p53 accompanied by downregulated PRMT5 and that forced expression of PRMT5 in *Fanca^−/−^* HSPCs prolonged oncogenic response and delayed leukemia development in irradiated recipient mice (Figure 4). Thus, these studies defined a compromised oncogene-induced arginine methylation of p53 by PRMT5 as one mechanism for the short-lived oncogenic stress response in FA HSPCs.

The apparent tumor-suppressor role of PRMT5 identified in this work is in contrast to a recent study of Li *et al.* who demonstrated that PRMT5 for the inhibition of p53-dependent tumor suppression in response to oncogenic insults [57]. We noted three major differences in the experimental systems between our studies and those of Li *et al*. Firstly, while the study by Li *et al*. shows that PRMT5 is required for lymphomagenesis driven by multiple oncogenes including cyclin D1, c-MYC, NOTCH1, and MLL-AF9, the authors employed the Cyclin D1 D1T286A oncogenic model for PRMT5-related mechanistic studies. On the other hand, we used the K-ras^G12D^ model. Secondary, all of the studies by Li *et al*. were performed in T-cell lymphoma/Leukemia, which is different from our myeloid leukemia model. Lastly, our mouse model is deficient in the FA DNA repair pathway. In addition to these differences, our results appear to be consistent to a recent report, which shows that over-expression of PRMT5 caused cell cycle arrest while PRMT5 depletion resulted in apoptosis [58]. In this context, our study reveals the multiple facets of PRMT5 response to oncogenic activation. The mechanism by which PRMT5 is deregulated in the FA-deficient cells is not known at the moment. However, our recent proteomic study with a *Fancd2* knock-in mouse model shows that Fancd2 interacts with the murine Prmt5 *in vivo* (data not shown). We speculate that the FA pathway may play a role in maintaining PRMT5 activity in response to oncogenic stress leading to increased p53 arginine methylation and prolonged oncogenic response.

In conclusion, the present study, using an innovative *in vivo* model integrating the stress-responsive *Gadd45β*-luciferase transgene and inducible oncogenes (LSL-K-ras^G12D^ and Myc^ER^), uncovers two novel and significant findings: (i) normal HSPCs utilize arginine methylation of p53 by PRMT5 to orchestrate long-lasting oncogenic response. (ii) FA deficiency leads to a complete reversal of the response pattern characterized by short-lived oncogenic stress response accompanied with aberrant oncogene-induced arginine methylation of p53 resulted from a progressive decline in PRMT5 in FA HSPCs. Our studies indicate that a dysregulated PRMT5 in FA HSPCs plays an important role in suppressing p53-dependent tumor surveillance and suggest that PRMT5 may be a putative therapeutic target in FA tumorigenesis.

## MATERIALS AND METHODS

### Mice and treatment

*Fanca^+/+^*, *Fanca^−/−^* and *Fancc^+/+^*, *Fancc^−/−^* mice were generated by interbreeding the heterozygous *Fanca^+/−^* or *Fancc^+/−^* mice [59, 60], respectively. Heterozygous *Fanca^+/−^* or *Fancc^+/−^* mice were also interbred with the heterozygous Luc-Gadd45β mice [40] to generate Luc-*Fanca^+/−^* and Luc-*Fancc^+/−^* mice. Luc-*Fanca^+/+^,* Luc-*Fanca^−/−^,* Luc-*Fancc^+/+^,* and Luc-*Fancc^−/−^* mice were generated by interbreeding the heterozygous Luc-*Fanca^+/−^* or Luc-*Fancc^+/−^* mice. Luc-LSL-K-Ras^G12D^/CreER-*Fanca^+/+^* and Luc-LSL-K-Ras^G12D^/CreER-*Fanca^−/−^* mice were generated by interbreeding heterozygous Luc-*Fanca^+/−^* mice with LSL-K-Ras^G12D^ mice [38] expressing a *CreER* transgene [61].

For Cre-mediated gene deletion, animals were injected i.p. with 100 μl of tamoxifen (20 mg/ml; Sigma-Aldrich, St. Louis, MO). Animals were maintained in the animal barrier facility at Cincinnati Children's Hospital Medical Center. All experimental procedures conducted in this study were approved by the Institutional Animal Care and Use Committee of Cincinnati Children's Hospital Medical Center.

### Flow cytometry

The lineage marker (Lin) mixture (BD Biosciences, San Jose, CA) for BM cells from treated or untreated mice included the following biotinylated antibodies: CD3ε (145-2C11), CD11b (M1/70), CD45R/B220 (RA3-6B2), mouse erythroid cells Ly-76 (Ter119), Ly6G and Ly-6C (RB6-8C5). Other conjugated antibodies (BD sciences, San Jose, CA) used for surface staining included: CD45.1 (A20), CD45.2 (A104), Sca1 (D7), c-kit (2B8). Biotinylated primary antibodies were detected by incubation of antibody coated cells with streptavidin-PerCP or FITC (BD Biosciences, San Jose, CA) in a two-step staining procedure.

For apoptosis staining, LSK cells were cultured in the presence of 4-OHT (125 mM; Sigma-Aldrich, St. Louis, MO) for 24 hours followed by Annexin V and 7AAD staining using BD ApoAlert Annexin V kit (BD Pharmingen, San Jose, CA) in accordance with the manufacturer's instruction. Apoptosis was analyzed by quantification of Annexin V-positive cell population by flow cytometry.

For cell cycle analysis, LSK cells were cultured in the presence of 4-OHT for 2 hours and released for indicated time intervals followed by staining with propidium iodide (PI) solution containing 1 mg/mL RNase A. In another set of cell cycle analysis, treated stained cells were fixed and permeabilized with Cytofix/Cytoperm buffer (BD Pharmingen, San Jose, CA) followed by intensive wash using Perm/Wash Buffer (BD Pharmingen, San Jose, CA). Cells were incubated with anti-mouse Ki67 antibody (BD Pharmingen, San Jose, CA) and Hochest 33342 (Sigma-Aldrich, St. Louis, MO) followed by Flow cytometric analysis.

### IVIS imaging

Subjected mice pre-treated with indicated chemicals were injected by an intraperitoneal route with a luciferin solution (15 mg/ml in PBS (150 mg/kg)) that is allowed to distribute in awake animals for about 5–15 minutes. The mice are placed into a clear plexiglass anesthesia box (2.5–3% isofluorane) that allows unimpeded visual monitoring of animals. After the mice are fully anesthetized, they are transferred from the box to the nose cones attached to the manifold in the imaging chamber. The first image is taken approximately 5 minutes after luciferin injection. Continue to take images at indicated time points after the injection using IVIS series pre-clinical *in vivo* Imaging systems (PerkinElmer, Santa Clara, CA). The bioluminescence image signals were quantified by measuring photon flux using LiveImage Pro. 2.0 software (Caliper Life Science, Hopkinton, MA) as previously reported [62]. Briefly, specific areas of the image were analyzed by creating regions of interest (ROI) and measured the average # of photo/second. After subtracting the signal background of luciferin injected animals at time 0, the net photon flux (p/s/cm^2^/sr) and the photon flux were normalized to the respective values obtained at different time points after Tamoxifen injection and were plotted versus days after injection. GraphPad Prism was subsequently used to generate graphs for this manuscript.

### Isolation of BM lineage-depleted cells

The femora and tibiae were harvested from the mice immediately after their sacrifice with CO_2_. Bone marrow (BM) cells were flushed from bones into Iscove's modified Dulbecco's medium (IMDM; Invitrogen, Grand Island, NY) containing 10% FCS, using a 21-guage needle and syringe. Low-density BM mononuclear cells (LDBMMNCs) were separated by Ficoll Hypaque density gradient (Sigma-Aldrich, St. Louis, MO) and washed with IMDM medium. LDBMMNCs were depleted of lineage-committed cells using a lineage cell depletion kit (Miltenyi Biotec, San Diego, CA) in accordance with the manufacturer's instruction.

### Colony-forming unit assay

LSK cells (Lin^−^Sca1^+^c-kit^+^ cells) were cultured with 4-OHT (125 mM; Sigma-Aldrich, St. Louis, MO) for 48 hours followed by plating in a 35-mm tissue culture dish in 4 mL of semisolid medium containing 3 mL of MethoCult M3134 (Stem Cell Technologies, Vancouver, BC, Canada) and the following growth factors: 100 ng/mL SCF, 10 ng/mL IL-3, 100 ng/mL GM-CSF, and 4 units/mL erythropoietin (Peprotech, Burlington, NC). On day 7 after plating, erythroid and myeloid colonies were enumerated. Hematopoietic clonal growth results were expressed as means (of triplicate plates) ± SD of three experiments.

### Quantitative PCR analysis

LSK cells were cultured in the presence of 4-OHT for 2 hours then released in fresh medium for the indicated time intervals. RNA was then extracted for qPCR analysis using primers listed in Table S1. Samples were normalized to the level of *GAPDH* mRNA.

### Viral expression vectors

The lentiviral vector expressing FLAG-tagged full-length FANCA was described previously [63]. The Myc-tagged full-length human PRMT5 cDNA was subcloned into lentiviral vector pLVX-IRES-GFP vector (Clontech, Mountain View, CA), using the gateway system (Life Technologies, Grand Island, NY). The MSCV-IRES-GFP-Myc^ER^ retroviral vector was a gift from Dr. Cleveland JL at St. Jude Children's Research Hospital [39]. The plasmids (10 μg each) were used to produce retroviral supernatant.

### Bone marrow transplantation

BM Lin^−^ cells isolated from either Luc-*Fanca^+/+^* or Luc-*Fanca^−/−^* mice were transduced with retroviral vector MSCV-IRES-eGFP-MycER [39]. 1,500 transduced *Fanca^+/+^* LSK cells and 3,000 *Fanca^−/−^* LSK cells, along with 3×10^5^ BM cells from congenic BoyJ mice, were subjected to bone marrow transplantation (BMT) into lethally irradiated BoyJ recipients. For BMT of Luc-LSL-K-Ras^G12D^/CreER cells, 1,000 LSK cells from Luc-LSL-K-Ras^G12D^/CreER-*Fanca^+/+^* mice or 2,000 LSK cells from Luc-LSL-K-Ras^G12D^/CreER-*Fanca^−/−^* mice, along with 3×10^5^ BM cells from congenic BoyJ mice, were used. Four months post BMT, donor-derived chimera was determined by Flow Cytometric analysis. The animals were then subjected to the indicated *in vivo* treatment and IVIS imaging.

### Preparation of cell extracts, immunoblotting and immunoprecipitation

To prepare protein lysates, sorted Lin^−^ cells were washed with ice-cold PBS, and resuspended in ice-cold lysis buffer containing 50 mmol/L Tris-HCL (pH 7.4), 0.1% NP40, and 1 mol/L NaCl supplemented with protease and phosphatase inhibitors [10 μg/mL aprotinin, 25 μg/mL leupeptin, 10 μg/mL pepstatin A, 2 mmol/L phenylmethylsulfonyl fluoride, 0.1 mol/L NaP_2_O_4_, 25 mmol/L NaF, and 2 mmol/L sodium orthovandate] for 30 min on ice. Cell debris was removed from the lysates by centrifuging them at 14,000 rpm for 30 min. Protein concentration was quantified by using Bio-Rad reagent and resolved on SDS-PAGE and transferred onto nitrocellulose membranes. Immunoblots were then incubated with primary antibodies specific for p53 (R&D, Minneapolis, MN), PRMT1, PRMT4, PRMT5 (Abcam, Cambridge, MA), phospho-CHK1 (Ser345), total CHK1, phospho-CHK2 (Thr68), total CHK2 (all from Cell Signaling Technology, Danvers, MA), or β-actin (Sigma-Aldrich, St. Louis, MO). Quantitative analysis was performed by utilizing ImageJ software (NIH).

For immunoprecipitation, protein A/G agarose beads (Santa Cruz Biotechnologies, Dallas, Texas) precleaned cell lysates were incubated with p53 antibody (R&D, Minneapolis, MN) by gentle rocking overnight at 4°C followed by incubation with protein A/G agarose beads for additional 1 hour at 4°C. Pellets were then washed with 500 ml of lysis buffer and resuspended with 20 ml of 4X SDS sample buffer. Immunoblots were then incubated with primary antibodies specific for phosphor-serine (Millipore, Billerica, MA), acetylated lysine (Abcam, Cambridge, MA), methylated lysine (Abcam, Cambridge, MA), or mono-methyl arginine (Cell Signaling, Danvers, MA) antibodies for 12 to 16 h at 4 degree. Signals were revealed after incubation with anti-mouse or anti-rabbit secondary antibodies.

### Statistics

Data were analyzed statistically using a Student's *t* test. The level of the statistical significance stated in the text was based on the *P value*s. *P* < 0.05 was considered statistically significant.

## SUPPLEMENTARY MATERIALS FIGURES AND TABLE


