# Supplementary material for: The Fanconi anemia pathway controls oncogenic response in hematopoietic stem and progenitor cells by regulating PRMT5-mediated p53 arginine methylation
Source: Oncotarget. 2016 Aug 5;7(37):60005–20. doi: 10.18632/oncotarget.11088 (PMC5312365; doi:10.18632/oncotarget.11088)
Supplement: Supplementary file 1 [file oncotarget-07-60005-s001.pdf]

The Fanconi anemia pathway controls oncogenic response in hematopoietic stem and progenitor cells by regulating PRMT5-mediated p53 arginine methylation

Supplementary Materials

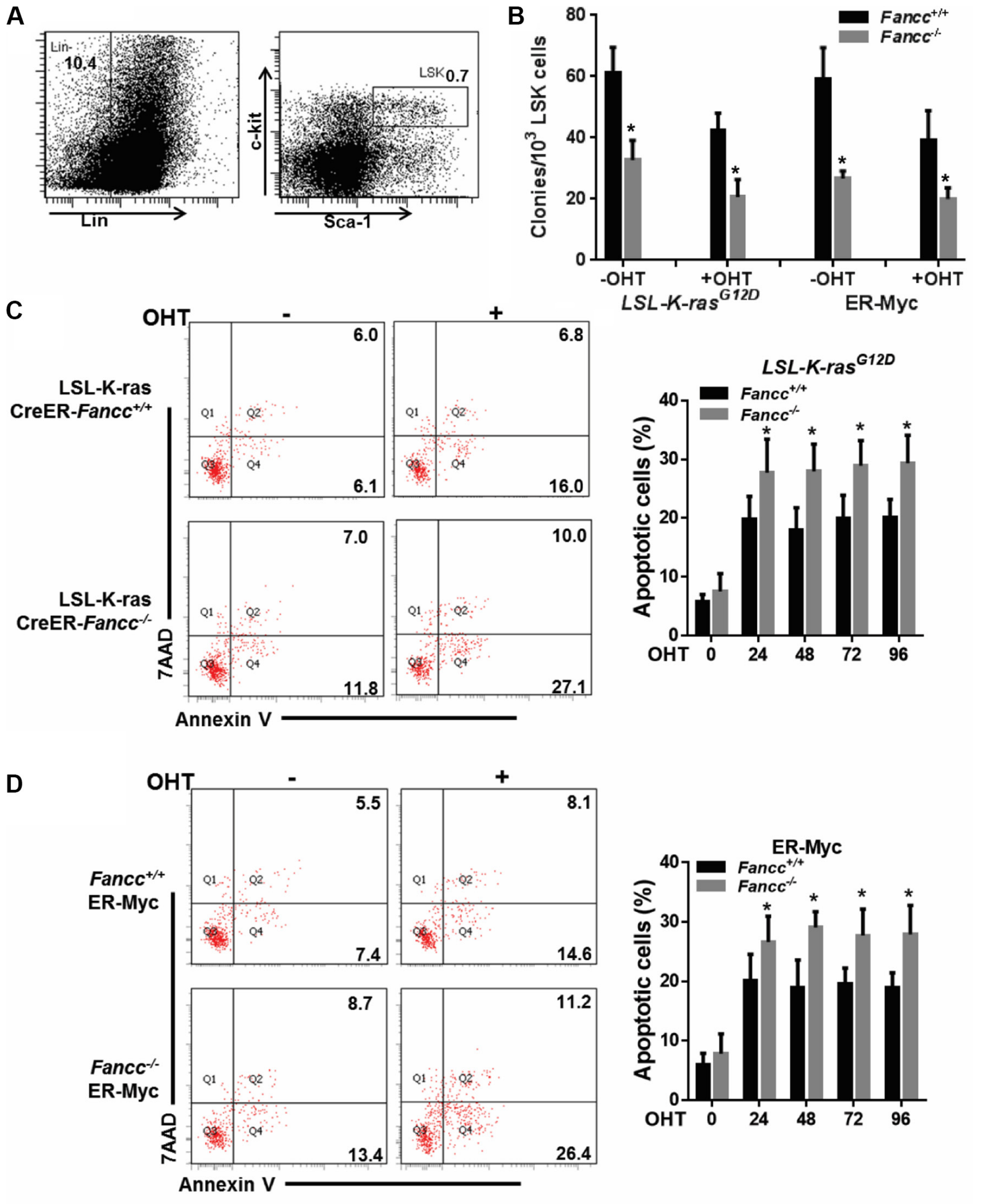

E

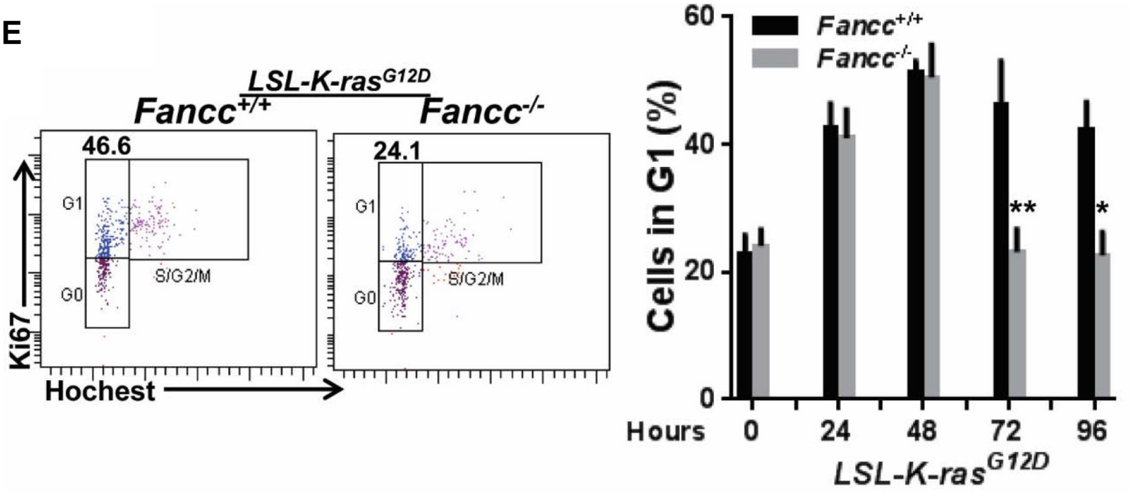

F

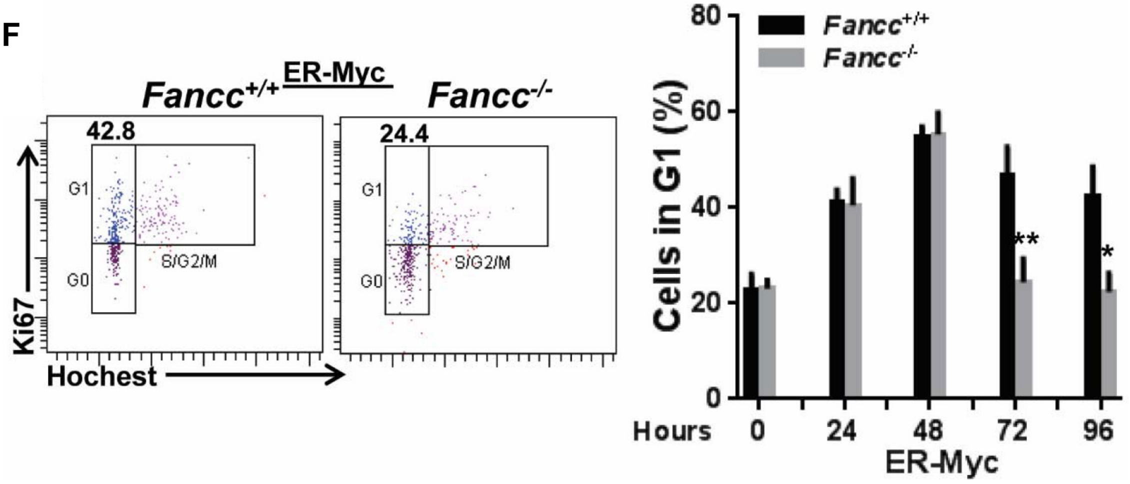

Supplementary Figure S1: *Fancc* deficiency induces a short-lived response to oncogenic stress *in vitro*. (**A**) Gating strategy for apoptosis and cell-cycle analysis. Low density bone marrow cells (LDBMCs) isolated from experimental mice were subjected to Flow cytometric analysis. (**B**) Oncogenic stress compromises colony formation capacity of FA HSPCs. LSK cells (Lin$^-$Sca1$^+$c-kit$^+$ cells) isolated from LSL-*Fancc*$^{+/+}$/K-ras/CreER and LSL-*Fancc*$^{-/-}$/K-ras/CreER mice, or retroviral vector MSCV-IRES-Myc^{ER} transduced LSK cells from *Fancc*$^{+/+}$ or *Fancc*$^{-/-}$ mice were *in vitro* culture in the presence of 4-OHT for 48 hours followed by plating in cytokine-supplemented methycellulose medium. Colonies were enumerated on day 7 after plating. Results are means ± standard deviation (SD) of 3 independent experiments (n = 9 per group). (**C**) K-ras activation induces apoptosis in FA HSCs. LSK cells (Lin$^-$Sca1$^+$c-kit$^+$ cells) isolated from LSL-*Fancc*$^{+/+}$/K-ras/CreER and LSL-*Fancc*$^{-/-}$/K-ras/CreER mice were subjected to Flow cytometric analysis for apoptosis by Annexin V/7AAD staining at different time points. Representative images (left) and quantification (right) were shown. Results are means ± standard deviation (SD) of 3 independent experiments (n = 6 per group). (**D**) Myc activation induces apoptosis in FA HSCs. Retroviral vector MSCV-IRES-Myc^{ER} transduced LSK cells from *Fancc*$^{+/+}$ or *Fancc*$^{-/-}$ mice were subjected to Flow cytometric analysis for apoptosis by Annexin V/7AAD staining. Representative images (left) and quantification (right) were shown. Results are means ± standard deviation (SD) of 3 independent experiments (n = 9 per group). (**E**) Activation of K-ras leads to short-lived G$_1$ arrest in FA cells. Cells described in (C) were subjected to cell cycle profiling by Hochest33324/Ki67 staining. Representative images (left) and quantification (right) were shown. Results are means ± standard deviation (SD) of 3 independent experiments (n = 6 per group). (**F**) Activation of Myc leads to short-lived G$_1$ arrest in FA cells. Cells described in (D) were subjected to cell cycle profiling by Hochest33324/Ki67 staining. Representative images (left) and quantification (right) were shown. Results are means ± standard deviation (SD) of 3 independent experiments (n = 9 per group).

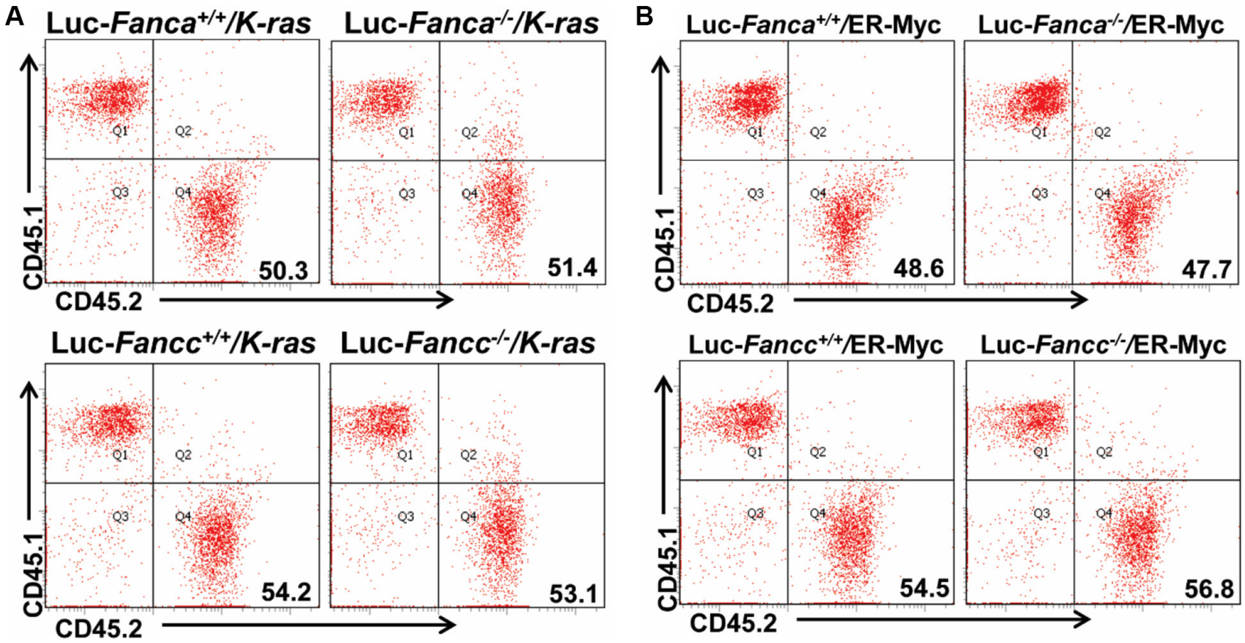

Supplementary Figure S2: Establishment of Luc-Fanc/Kras or Luc-Fanc/MycER chimera. (**A**) 1,000 LSK cells from Luc-LSL-K-ras/CreER-*Fanca*$^{+/+}$ or 2,000 LSK cell from Luc-LSL-K-ras/CreER-*Fanca*$^{-/-}$ mice along with 3×10^5 BM cells from congenic BoyJ mice were transplanted into lethally irradiated BoyJ recipients. Donor-derived hematopoiesis was determined by Flow Cytometery at 4-month post BMT (Upper). Similar experiments were conducted using Luc-LSL-K-ras/CreER-*Fancc*$^{+/+}$ or Luc-LSL-K-ras/CreER-*Fancc*$^{-/-}$ cells (Lower). (**B**) LSK cells from Luc-*Fanca*$^{+/+}$ or Luc-*Fanca*$^{-/-}$ mice were transduced with retroviral vector MSCV-IRES-Myc^{ER}. 1,500 transduced Luc-*Fanca*$^{+/+}$ cells or 3,000 transduced Luc-*Fanca*$^{-/-}$ cells along with 3×10^5 BM cells from congenic BoyJ mice were transplanted into lethally irradiated BoyJ recipients. Donor-derived hematopoiesis was determined by Flow Cytometery at 4-month post BMT (Upper). Similar experiments were conducted using Luc-*Fancc*$^{+/+}$ or Luc-*Fancc*$^{-/-}$ cells (Lower).

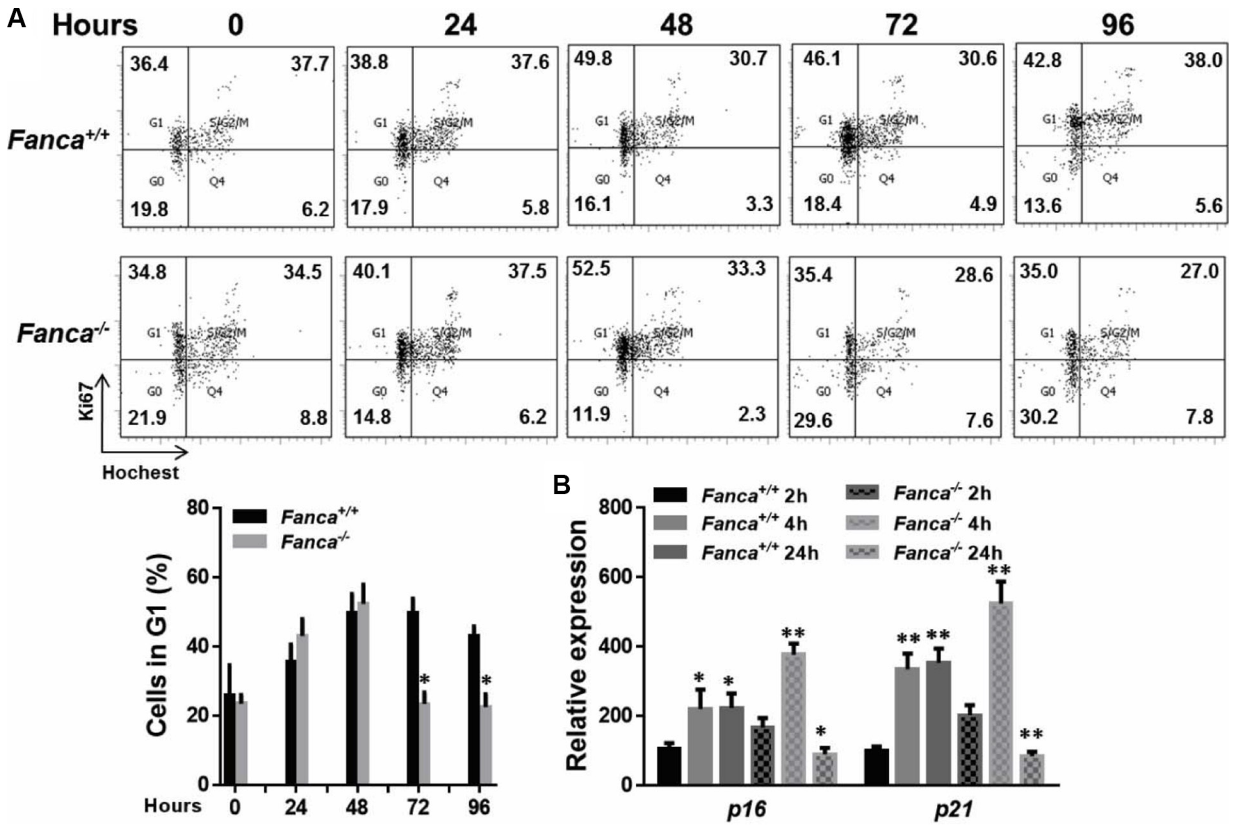

Supplementary Figure S3: Altered oncogenic response in *Fanca*$^{-/-}$ MEF cells. (**A**) Activation of K-ras leads to short-lived G_1 arrest in *Fanca*$^{-/-}$ MEF cells. Primary mouse embryonic fibroblasts (MEFs) isolated from Luc-LSL-K-ras/CreER-*Fanca*$^{+/+}$ and Luc-LSL-K-ras/CreER-*Fanca*$^{-/-}$ mice, were cultured in the presence of 4-OHT for 2 hours then released in fresh medium for the indicated time intervals, followed by cell cycle profiling by Hochest33324/Ki67 staining. Representative flow plots (left) and quantification (right) are shown. Results are means ± standard deviation (SD) of 3 independent experiments (n = 6 per group). (**B**) Expression of cell cycle regulators. RNA extracted from MEFs described in (A) at different time points were subjected to qPCR analysis using primers for *p16* and *p21*. Levels of the expression in each sample were normalized to the level of *GAPDH* mRNA, and the expression levels of the *Fanca*$^{+/+}$ samples at 2 h were normalized as 100.

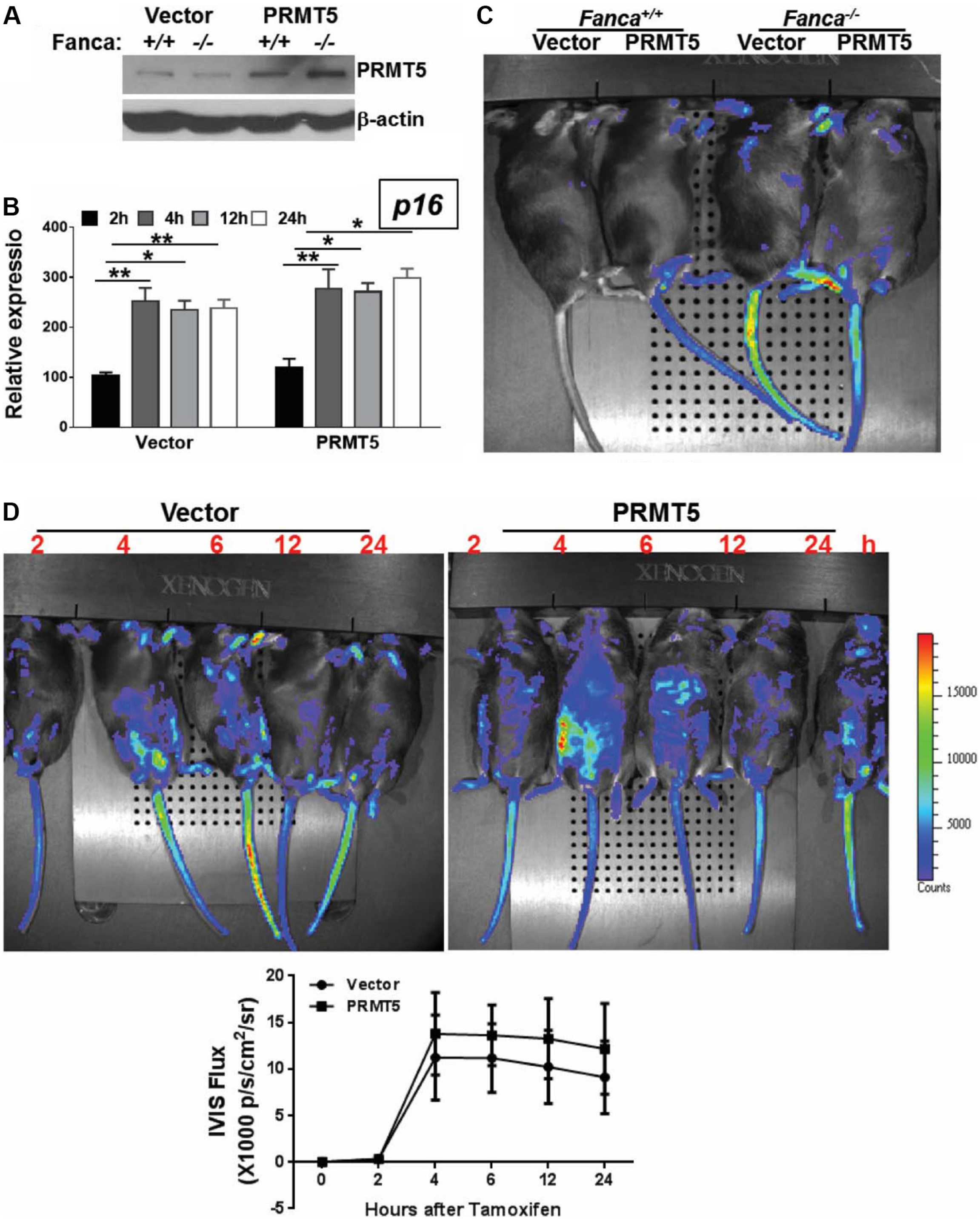

Supplementary Figure S4: Ectopic expression of PRMT5 augments stress response. (**A**) Ectopic expression of PRMT5 in *Fanca*$^{+/+}$ and *Fanca*$^{-/-}$ cells. BM Lin$^-$ cells from Luc-LSL-*Fanca*$^{+/+}$/K-ras/CreER or Luc-LSL-*Fanca*$^{-/-}$/K-ras/CreER mice were transduced with lentiviral vector expressing PRMT5. Sorted Venus$^+$ cells were then subjected to immunoblotting using antibodies against for PRMT5 or β-actin. (**B**) Forced expression of PRMT5 does not affect *p16* expression in WT cells. LSK cells from WT mice were transduced with lentiviral vector expressing PRMT5. Sorted Venus$^+$ cells were cultured in the presence of 4-OHT for 2, 4, 12 and 24 hours followed by RNA extraction and qPCR analysis using primers for *p16.* Samples were normalized to the level of *GAPDH* mRNA, and the expression level of the sample at 2h was normalized as 100. (**C**) Minimum bioluminescence of Luc mice without Tamoxifen injection. LSK cells from Luc-LSL-*Fanca*$^{+/+}$/K-ras/CreER mice were transduced with lentiviral vector expressing PRMT5. Sorted Venus$^+$ cells were used for BMT to lethally irradiated BoyJ recipients. 4-month post BMT, live images of the recipient mice were taken right before tamoxifen injection. Luminescence scale is in p/s/cm2/sr. (**D**) Ectopic expression of PRMT5 augmented K-ras-induced luciferase expression in WT donor cells. Single dose of 4-OHT were administrated to the recipient mice described in (C) and live images were taken at indicated time after 4-OHT induction. The bioluminescent image signals were quantified using LiveImage Pro. 2.0 software. Results are means ± standard deviation (SD) of 3 independent experiments (*n* = 6 per group).

Supplementary Table S1: Primers used for qPCR

Gene	Forward	Reverse
p15	AGATCCCAACGCCCTGAACCG	TGCTCTTCAGCCAAGTCTACC
p16	CGAACTCTTTCGGTCGTACCC	TTGAGCAGAAGAGCTGCTACG
p19	CGGAATCCTGGACCAGGTG	ACCAGCGTGTCCAGGAAGC
p21	GGCCCGGAACATCTCAGG	AAATCTGTCAGGCTGGTCTGC
